# Association Between the Lifestyle Inflammation Score and Gestational Diabetes Mellitus and Postpartum Glucose Metabolism Alterations

**DOI:** 10.3390/nu17233717

**Published:** 2025-11-27

**Authors:** Mónica L. Ruiz-Martínez, Rita A. Gómez-Díaz, Adriana Leticia Valdez-González, Luz Angélica Ramírez-García, Gabriela Eridani Acevedo-Rodríguez, María Teresa Ramos-Cervantes, Mary Flor Díaz-Velázquez, Marco Antonio Morales-Pérez, Rafael Mondragón-González, Niels H. Wacher

**Affiliations:** 1 Unidad de Investigación Médica en Epidemiología Clínica UMAE HE CMN SXXI, Instituto Mexicano del Seguro Social (IMSS), Av. Cuauhtémoc 330, Mexico City 06720, Mexico; ausie23@gmail.com (M.L.R.-M.); morales.marcoant@gmail.com (M.A.M.-P.);; 2 Servicio de Endocrinología, Unidad Médica de Alta Especialidad, Hospital de Gineco-Obstetricia No. 4 “Luis Castelazo Ayala”, Instituto Mexicano del Seguro Social (IMSS), Río de la Magdalena 289, Mexico City 01090, Mexico; 3 Centro Médico Nacional “La Raza”, Dr. Víctor Manuel Espinosa de los Reyes Sánchez, Instituto Mexicano del Seguro Social (IMSS), Eje Vial 1 Poniente, Mexico City 02990, Mexico; 4 Unidad Médica de Alta Especialidad, Hospital de Gineco-Obstetricia No. 3, Centro Médico Nacional “La Raza”, Dr. Víctor Manuel Espinosa de los Reyes Sánchez, Instituto Mexicano del Seguro Social (IMSS), Avenida Vallejo S/N Esq. Antonio Valeriano S/N, Col. La Raza, Mexico City 02990, Mexico

**Keywords:** lifestyle inflammation score, gestational diabetes mellitus, glucose metabolism alterations, postpartum, inflammation, risk factors

## Abstract

**Background/Objectives:** This study aimed to assess the associations between the Lifestyle Inflammation Score (LIS) and gestational diabetes mellitus (GDM), and glucose metabolism alterations (GMA) postpartum. **Methods:** A secondary analysis was conducted on 378 pregnant women at the end of pregnancy. Anthropometric, clinical, biochemical, and dietary parameters were evaluated. Postpartum reclassification was based on fasting serum glucose (>100 mg/dL), HbA1c (>5.7%), and/or a 2-h oral glucose tolerance test (>140 mg/dL). The LIS was calculated using a proxy index including smoking status, physical activity, and pregestational BMI, applying the beta coefficient from the original LIS model. Tertiles were created, with T3 indicating the highest level of proinflammatory exposure. Statistical analyses included Kruskal–Wallis, one-way ANOVA, linear-by-linear association, and multivariate logistic regression, adjusted for family history, gestational weight gain, carbonylated proteins, and adiponectin to evaluate associations between LIS and GDM, and adjusted for pharmacological treatment, gestational weight gain, and breastfeeding for LIS and GMA. **Results:** Higher LIS values were more common among women with GDM (T1 = 45.9%, T2 = 62.2%, T3 = 74.8%, *p* < 0.001) and among those with GMA (T1 = 34.4%, T2 = 45.6%, T3 = 53.7%, *p* = 0.019). Compared with the lowest tertile, the highest tertile of LIS was associated with greater odds of GDM (OR 3.72; 95% CI: 1.19–11.64, *p* = 0.024) and GMA (OR 2.69; 95% CI: 1.25–5.76, *p* = 0.011). **Conclusions:** A more proinflammatory lifestyle, as reflected by a higher LIS, increases the risk of progression to GDM and later to GMA.

## 1. Introduction

Poor diet quality, obesity, physical inactivity, smoking, and excessive alcohol intake are all modifiable lifestyle factors that contribute to low-grade inflammation and have been shown to increase the risk of gestational diabetes mellitus (GDM) and glucose metabolism alterations (GMA) in the postpartum period [1,2,3,4,5].

Among the key risk factors for progression from GDM to type 2 diabetes (T2D) are (1) greater severity of GDM (e.g., insulin use or higher blood glucose levels); (2) higher body mass index (BMI) at any stage—pregestational, during pregnancy, or later in life; (3) unhealthy dietary habits; (4) pregnancy complications beyond GDM (such as stillbirth, gestational hypertension, or cesarean section); (5) low levels of physical activity; and (6) non-European ancestry. Conversely, protective lifestyle factors include: (1) regular physical activity during the perinatal and postpartum periods; (2) breastfeeding; and (3) adherence to a healthy dietary pattern [6]. A meta-analysis demonstrated that lifestyle interventions comprising dietary changes and/or physical activity, implemented within three years postpartum, significantly reduced the risk of developing T2D by 43% [7].

Exercise is one of the main lifestyle factors that decreases systemic inflammation. It is associated with lower levels of inflammatory biomarkers such as C-reactive protein (CRP), interleukin-6 (IL-6), and tumor necrosis factor-alpha (TNF-α), through multiple mechanisms, including a reduction in visceral adiposity, an increase in muscle mass, improved insulin sensitivity, increased glucose uptake, and strengthened antioxidant defenses [8].

The Lifestyle Inflammation Score (LIS) was developed to quantify the cumulative pro- or anti-inflammatory potential of four major lifestyle characteristics: smoking status, BMI, alcohol intake, and physical activity. This score captures the combined contribution of these factors to systemic inflammation [9].

Previous studies have shown that higher LIS values are associated with increased risk of type 2 diabetes, metabolic syndrome, obesity, and colorectal adenoma and higher all-cause, cancer-specific, and cardiovascular disease-specific mortality [3,10,11,12,13,14].

Low-grade inflammation (LGI) is a critical mechanism contributing to both the onset of GDM and its progression to T2D. Proinflammatory cytokines such as tumor necrosis factor-α (TNF-α), interleukin-6, and galectin-3, induce insulin resistance [15]. The level of TNF-α has been reported to be higher in patients with T2D. Additionally, TNF-α, IL-6, and leptin have been associated with the risk of GDM [15,16,17]. Unhealthy lifestyle factors such as poor dietary habits, physical inactivity, obesity, and aging promote LGI, which in turn drives insulin resistance and the pathogenesis of T2D [18]. GDM is characterized by insulin resistance, dysregulated adipokine levels, excessive lipolysis, heightened oxidative stress, and systemic inflammation, all of which increase the likelihood of developing T2D later in life [19,20].

Among the adipokines affected by this process, adiponectin is reduced in GDM (hypoadiponectinemia). Lower adiponectin levels are associated with increased insulin resistance, excessive gestational weight gain, and a higher risk of postpartum GMA [21,22]. Leptin, another dysregulated adipokine, tends to increase (hyperleptinemia) resulting from central leptin resistance, which is related to obesity, insulin resistance, and inflammation in both GDM and T2D [23,24].

Oxidative stress also plays a central role in the development of both GDM and T2D. It exacerbates inflammatory responses, and chronic oxidative stress may trigger persistent low-grade inflammation [20,25]. Carbonylated proteins, which are markers of protein oxidation, have been linked to T2D and its complications, which are caused by hyperglycemia and increased reactive oxygen species [26].

Despite the well-documented role of inflammation in metabolic disorders, no previous study has evaluated the association between the LIS, GDM, and GMA.

It is essential to assess whether the combination of hazardous lifestyle inflammatory components that have been proven to affect a woman during pregnancy and postpartum could potentiate the development of GDM and later to GMA, which have significant adverse health consequences.

The present study, therefore, aimed to assess the associations between the LIS and the risk of developing GDM during pregnancy and GMA in the postpartum period.

## 2. Materials and Methods

### 2.1. Study Design and Participants

This was a secondary analysis of data from pregnant women (*n* = 378) who participated in the original study “Effect of an educational intervention for the prevention and management of overweight and obesity in the first 18 months of life of the mother and child with GDM in beneficiaries of the Mexican Institute of Social Security (IMSS)” (registration number R-2018-785-079; ClinicalTrials.gov identifier: NCT04493177).

With the main objective of implementing and evaluating an educational intervention to prevent and manage overweight and obesity in the first 18 months of life for both mother and children with GDM, the inclusion criteria were women aged 18 to 40 years in the last trimester of pregnancy with or without GDM, the exclusion criteria were patients with chronic kidney disease, hepatic or cardiac disease, hypertensive diseases of pregnancy, severe pregnancy complications (diabetic ketoacidosis, coma, hypovolemic shock), mothers with type 1 diabetes, type 2 diabetes, maturity-onset diabetes of the young, or secondary diabetes.

The elimination criteria were when the newborn required advanced neonatal resuscitation, remained in the intensive care unit for five days or more, had congenital malformations, or was stillborn.

Participants provided informed re-consent for this analysis (registration R-2022-785-057). The inclusion criteria were as follows: women aged 18–40 years, with full-term singleton pregnancies confirmed by ultrasound, who delivered at two IMSS specialty hospitals in Mexico City, provided informed consent, and who attended the scheduled postpartum visits.

Women were excluded (*n* = 487) if they did not attend postpartum follow-up visits or were taking medications that altered glucose metabolism.

There were no statistically significant differences between the patients in the original cohort and the study participants in terms of smoking status, physical activity, or prepregnancy BMI. There were statistically significant differences in alcohol consumption, although they were not clinically important. Both groups consumed small amounts of alcohol, (the median for the included participants was 0.20 g (0–0.29 g) of ethanol, whereas it was 0.04 g (0–0.29 g), *p* = 0.020 for the excluded patients.

All participants had detailed clinical histories and underwent standardized assessments of anthropometric, biochemical, and dietary parameters.

### 2.2. Anthropometric and Clinical Measurements

Body weight, height, and BMI (pregestational, at the end of pregnancy, and postpartum) were calculated as weight (kg)/height (m^2^) and categorized as follows: normal: <25 kg/m^2^; overweight: 25–29.99 kg/m^2^; and obese: ≥30 kg/m^2^. Body fat percentage was measured using plicometry (skinfold thickness) with a Lange caliper, calculated using the Durnin and Womersley formula. Waist and hip circumferences were also recorded. Gestational weight gain was defined as the difference between pregestational weight and weight at the end of pregnancy [27,28].

Blood pressure was measured using standardized procedures in accordance with international guidelines [27].

### 2.3. Lifestyle Factors

Physical activity was assessed using the International Physical Activity Questionnaire (IPAQ) and categorized into three levels: physically inactive, moderately active, or highly active [29].

Smoking status was determined at the end of pregnancy and categorized as active smoker or non-smoker.

### 2.4. Lifestyle Inflammation Score (LIS)

Byrd et al. [9] constructed the original LIS. It includes four lifestyle components, which are alcohol consumption, physical activity, current smoking status, and an overweight or obese BMI [9]. (Table 1).

The LIS of Byrd et al. [9], did not consider a population that did not consume alcohol or women already at risk of GDM and GMA. We decided to use a proxy that included three components: pregestational BMI, physical activity, and smoking status. Alcohol intake was excluded because consumption was negligible in this population, with a median of 0.20 g (0–0.65 g) of ethanol per day. The same *β* coefficients were considered as those described by Byrd et al. [9].

All lifestyle components were dummy variables, coded as 1 for the non-referent category and 0 for the referent category. Each component was multiplied by its corresponding *β* coefficient from the original LIS model, and the weighted components were totaled to obtain the final score. The participants were then divided into tertiles (T), with T3 representing the highest level of proinflammatory exposure.

For example, if a woman performed moderate physical activity, we would have −0.18 multiplied by 1; if she were a current smoker, we would have 0.50 multiplied by 1; and if she had an overweight prepregnancy BMI, we would have 0.89 multiplied by 1. The sum of the *β* coefficients give us a score. Moreover, the total score was divided into tertiles to determine the highest or lowest LIS.

### 2.5. Biochemical Analyses

Serum samples were collected after an 8–12-h overnight fast at the end of pregnancy and postpartum.

Glucose and triglycerides levels were measured using the Beckman Coulter^®^ Unicel DxC 600 Synchron clinical system (Beckman Coulter, Miami, FL, USA). Insulin levels were determined using the Beckman Coulter^®^ Access 2 system. HbA1c was analyzed from whole blood using high-performance liquid chromatography (HPLC) with the Variant II Turbo 2.0 kit (Bio-Rad, Hercules, CA, USA). Low-density lipoprotein (LDL) cholesterol was calculated with the Martin–Hopkins method [30]. HOMA-IR was computed as fasting serum glucose (mg/dL) × fasting insulin (μU/mL)/405 [31]. Carbonylated proteins were quantified from serum using colorimetric assays, and adiponectin and leptin were determined using commercial ELISA kits (R&D Systems, Minneapolis, MN, USA) [32].

### 2.6. Postpartum Reclassification

Only women who underwent biochemical reassessment after the third month postpartum were included (*n* = 378). The participants were categorized as follows:

1. Glucose metabolism alterations (GMA): fasting serum glucose (FSG) > 100 mg/dL, and/or 2-h OGTT glucose > 140 mg/dL, and/or HbA1c > 5.7%.
2. Normal glycemia: FSG < 100 mg/dL and 2-h OGTT glucose < 140 mg/dL [33].

### 2.7. Dietary Assessment

Dietary intake was assessed using a validated Food Frequency Questionnaire (FFQ) designed explicitly for the Mexican population [34]. The FFQ was administered on the same day as the OGTT. Nutritional composition was analyzed with SNUT Nutritional Analysis Software 3.0, and macronutrients were expressed as a percentage of total energy intake.

All anthropometric and biochemical measurements were conducted by trained health professionals (general practitioners, nutritionists, and nurses) following standardized protocols.

### 2.8. Statistical Analysis

Comparisons across the three LIS tertiles were performed:

Kruskal–Wallis or one-way ANOVA was used for quantitative variables (depending on distribution assessed by the Kolmogorov–Smirnov test). A linear-by-linear association test was performed for categorical variables. To evaluate the effect size, *η*^2^ for continuous variables and Cramer’s V for categorical variables were used. The negative *η*^2^ were truncated to <0.001.

Two multivariate logistic regression analyses were conducted: (1) LIS and GDM, adjusted for family history of diabetes, gestational weight gain, carbonylated proteins, and adiponectin. (2) LIS and GMA, adjusted for pharmacological treatment during pregnancy, gestational weight gain, and exclusive breastfeeding.

Statistical significance was set at *p* < 0.05, and analyses were performed using SPSS version 21 (SPSS Inc., Chicago, IL, USA).

## 3. Results

### 3.1. Results (Part 1)

The total population of pregnant women (*n* = 378) was analyzed, 235 with gestational diabetes mellitus (GDM) and 143 without. Clinical, anthropometric, biochemical, and dietary parameters were evaluated according to tertiles of the Lifestyle Inflammation Score (LIS). The distribution of these characteristics is presented in Table 2, Table 3 and Table 4.

Women in the highest tertile of LIS (T3) exhibited an overall poorer clinical and anthropometric profile. They had higher BMI at all stages (prepregnancy, end of pregnancy, and postpartum), greater body fat and waist-to-hip ratio, and higher postpartum blood pressure, while also presenting lower pregnancy weight gain. In addition, physical inactivity and smoking were more frequent in T3. The variables with the largest effect sizes were related to BMI, percentage of fat mass, and smoking status (Table 2).

Biochemically, higher LIS aligned with greater insulin resistance and a more adverse lipid–glycemic profile, particularly postpartum (Table 3).

At the end of pregnancy, women in the highest tertile showed higher fasting insulin, HbA1c, HOMA-IR, adiponectin, and carbonylated protein levels compared with those in the lower tertiles. These differences were more pronounced postpartum, with higher fasting glucose, triglycerides, LDL cholesterol, uric acid, fasting insulin, HbA1c, and HOMA-IR, in addition to lower HDL cholesterol in T3. All the biochemical variables had a small effect size.

These results indicate a clear association between a proinflammatory lifestyle and poorer metabolic control both during and after pregnancy.

Among dietary parameters, only total energy intake differed significantly across LIS tertiles, with lower intake observed in women in the highest tertile. The macronutrient distribution (% of total energy from proteins, carbohydrates, and fats) did not show significant variation between groups. All dietary parameters had a small effect size (Table 4).

Women with GDM were more frequently represented in the highest LIS tertile (T1 = 45.9%, T2 = 62.2%, T3 = 74.8%; *p* < 0.001, linear-by-linear association test).

In the multivariate logistic regression model, women in the highest LIS tertile had a significantly higher likelihood of GDM compared with those in the lowest tertile (OR 3.723; 95% CI: 1.191–11.640; *p* = 0.024) (Table 5).

### 3.2. Results (Part 2)

Of the 235 women with GDM, 102 (43.4%) developed glucose metabolism alterations (GMA) postpartum, identified by elevated fasting glucose and/or 2-h OGTT results. Among the 143 women without GDM, 11 (7.6%) presented elevated HbA1c levels postpartum. Together, these 246 women were classified as at risk for GMA and analyzed accordingly.

#### 3.2.1. Clinical and Anthropometric Findings

Women in the highest tertile of LIS (T3) had obesity at all stages—before pregnancy, at the end of pregnancy, and postpartum—whereas women in the lowest tertile (T1) maintained normal weight before and after pregnancy.

Women in T3 were also less physically active, smoked more, required more pharmacological treatment during pregnancy, and had higher postpartum fat mass, waist-to-hip ratio, and blood pressure, but gained less weight during pregnancy (Table 6).

Regarding magnitude, effect size estimates showed that pregestational and postpartum BMI, body fat percentage, and smoking status were most strongly associated with higher LIS, while other clinical variables displayed small effect sizes. These findings reinforce the role of a proinflammatory lifestyle in worsening metabolic and cardiovascular markers among women at risk for GMA.

#### 3.2.2. Biochemical Parameters

At the end of pregnancy, the only biochemical variable significantly different across LIS tertiles was uric acid (*p* = 0.048). However, in the postpartum period, most metabolic markers were adversely affected among women with higher LIS (Table 7).

Postpartum results demonstrated a clear gradient of metabolic impairment associated with higher LIS. Women in the highest tertile exhibited higher fasting and 2-h glucose levels, elevated triglycerides and LDL cholesterol, greater uric acid concentrations, and higher fasting and 2-h post-OGTT insulin levels, along with increased HOMA-IR. HDL cholesterol tended to be lower in T3. Overall, these findings indicate that a higher pro-inflammatory lifestyle is accompanied by more adverse metabolic markers in the postpartum period.

#### 3.2.3. Dietary Parameters

No significant differences were found in total energy intake or macronutrient distribution among LIS tertiles in the postpartum period (Table 8).

Total energy intake and macronutrient distribution in the postpartum period were similar across LIS tertiles. Therefore, the observed metabolic deterioration associated with higher LIS does not appear to be explained by dietary composition but rather by other lifestyle elements included in the score, particularly adiposity, smoking, and reduced physical activity.

#### 3.2.4. Association Between LIS and Postpartum Glucose Metabolism Alterations

Women with GMA were more likely to belong to the highest LIS tertile: T1 = 34.4%, T2 = 45.6%, and T3 = 53.7% (*p* = 0.019), as determined by the linear-by-linear association test.

In the multivariate logistic regression adjusted for pharmacological treatment during pregnancy, gestational weight gain, and exclusive breastfeeding, the highest LIS tertile was associated with a significantly increased risk of GMA (Table 9).

After adjusting for number of months postpartum, and using the 3–6 month postpartum as the reference, undergoing the OGTT at months 9–12 was associated with higher odds of GMA (OR 2.701, CI 95%: 1.080–6.756, *p* = 0.034), and undergoing it at 15 months or more postpartum was also associated with increased odds (OR 2.429, CI 95%: 1.182–4.992, *p* = 0.016).

Although we found an association between the higher LIS tertile and the risk of GMA, LIS has limited predictive performance, as we lack validation measurements, such as inflammation biomarkers.

These findings demonstrate a clear relationship between a higher Lifestyle Inflammation Score and increased risk of both gestational diabetes mellitus and postpartum glucose metabolism alterations. These associations persisted after adjustment for multiple confounding factors, underscoring the clinical value of the LIS as a potential screening and risk stratification tool in obstetric populations.

## 4. Discussion

This study is the first to assess the associations between the Lifestyle Inflammation Score (LIS), gestational diabetes mellitus (GDM), and postpartum glucose metabolism alterations (GMA). Our findings demonstrate that women in the highest LIS tertile were significantly more likely to develop GDM during pregnancy and GMA after delivery. These results suggest that the LIS could serve as an efficient, low-cost screening tool to identify women at increased risk of metabolic complications during and after pregnancy.

Previous research has shown that higher LIS values are associated with various chronic conditions, including type 2 diabetes (T2D), metabolic syndrome, obesity, and incident colorectal adenoma Additionally, a combination of higher LIS and a higher dietary inflammatory score has been associated with an increased risk of T2D [3,4,10,11,12,13,14]. The current study extends this evidence by linking a proinflammatory lifestyle to the metabolic continuum from GDM to GMA, a progression known to predispose women to T2D later in life.

### 4.1. Lifestyle and Anthropometric Factors

Women with higher LIS consistently exhibited obesity across all stages—prepregnancy, pregnancy, and postpartum. This finding aligns with established evidence indicating that obesity is one of the strongest risk factors for progression from GDM to GMA [6]. Even though women in the highest LIS tertile gained less weight during pregnancy and consumed less total energy, they began pregnancy with a higher BMI and remained obese afterward. This may explain the observed elevations in adiponectin levels, as gestational weight gain is inversely associated with adiponectin [21]. However, they probably also had lower energy intake and less pregnancy weight gain because they had restricted their diet for medical reasons or because of the severity of GDM.

Each one-unit increase in BMI between the pre-gestational and postpartum periods has been linked to an 18% increase in the risk of developing T2D, emphasizing the importance of effective weight management after GDM and throughout life [6,35]. Evidence also shows that postpartum lifestyle interventions—including diet and physical activity—significantly reduce the risk of both GDM recurrence and T2D development [1,5].

In our study, women in the highest LIS tertile had poorer body composition, with more than 10% higher total body fat and greater visceral adiposity (reflected by the waist-to-hip ratio). Visceral fat produces proinflammatory adipokines and has been positively associated with low-grade inflammation in individuals with recently diagnosed T2D, where hyperinsulinemia is a key mediator of this relationship [36]. Although the placenta can produce a series of proinflammatory cytokines during pregnancy, the primary source of cytokines is adipose tissue. TNF-α levels are inversely correlated with insulin sensitivity, while IL-6 and IL-1β inhibit insulin receptor substrate 1 (IRS-1); moreover, elevated levels of leptin in women with pregestational obesity contribute to insulin resistance [37,38,39]. All of these mechanisms suggest that excessive weight gain during pregnancy predisposes individuals to the development of GDM.

This pattern was also evident in our findings: women with higher LIS showed higher fasting insulin levels, 2-h post-OGTT insulin levels, and HOMA-IR, along with a greater prevalence of both GDM and GMA.

### 4.2. Metabolic Severity and Treatment

The severity of GDM, reflected by the need for pharmacological treatment, especially insulin, is a known predictor of progression to postpartum glucose dysregulation [6]. Women with higher LIS in our study required more pharmacological intervention during pregnancy, supporting this link between lifestyle-related inflammation and clinical severity.

### 4.3. Physical Activity and Smoking

Physical inactivity was a defining feature of women in the higher LIS tertiles. Physical activity is known to reduce systemic inflammation through the release of myokines and to improve insulin sensitivity via several mechanisms, including reducing visceral fat, enhancing muscle mass, promoting fat oxidation, enhancing glucose uptake, and lowering oxidative stress [8,40]. The inverse relationship between activity and LIS observed here reinforces the anti-inflammatory potential of exercise in metabolic health.

Smoking was also more prevalent among women in the highest LIS tertile. Maternal smoking during pregnancy is a major preventable risk factor for both maternal and neonatal morbidity. It restricts oxygen and nutrient delivery to the fetus, impairs organ development, and has adverse effects on the long-term health of offspring [41]. The evidence regarding smoking and GDM is mixed—some studies report no association, whereas others identify smoking as an independent risk factor for GDM and T2D [42,43,44,45,46].

Mechanistically, smoking increases visceral fat accumulation, promotes low-grade inflammation, reduces insulin sensitivity, and impairs β-cell function [36,46]. Our results are consistent with these findings: smoking contributes to a more proinflammatory lifestyle pattern, reflected in higher LISs and adverse metabolic outcomes.

### 4.4. Dietary Factors and Oxidative Stress

Although diet is a central component of lifestyle and plays a critical role in preventing both GDM and T2D, we did not observe statistically significant differences in macronutrient intake across LIS tertiles. This may be due to the quantitative nature of the FFQ, which captures nutrient amounts but not qualitative dietary patterns or food-based inflammatory potential. A dietary pattern analysis, such as the Dietary Inflammatory Index, might better capture these relationships [1,2,11,41].

Leptin levels were comparable across tertiles in the total population, possibly because women with the highest LIS gained less gestational weight, thereby lowering insulin resistance and leptin resistance [47]. In contrast, the subgroup analysis of women at risk for GMA revealed that leptin differences were not statistically significant, likely due to limited sample size [23]. The comparison of leptin across groups had a small effect size (*f* = 0.17) and yielded 65% power; therefore, the analysis may be susceptible to a type II error, as the study might not detect subtle differences between groups.

As expected, carbonylated protein levels, a marker of oxidative stress, were higher in women with higher LIS, which is consistent with the adverse metabolic and inflammatory milieu [19,25].

However, in the GDM subgroup, the magnitude of the group differences was small (*f* = 0.14). The analysis had approximately 51% power to detect an effect of this size. This level of power is below the commonly accepted threshold for adequate sensitivity, indicating that the study may have been insufficiently powered to detect subtle differences in protein carbonylation across groups. Therefore, the null finding should be interpreted as inconclusive rather than confirmatory.

Higher pre-pregnancy BMI, lower levels of physical activity, and smoking increase LGI, which in turn enhances insulin resistance; both mechanisms are key driver components of GDM and GMA. This is evident in the present study, where higher inflammation, as represented by a higher LIS tertile, was associated with a greater percentage of GDM and GMA [8,18,36,40,46].

## 5. Strengths and Limitations

### 5.1. Strengths

1. Our findings suggest that the LIS could identify women at risk of developing GDM and GMA, but more prospective studies with sensitivity and specificity analyses, discrimination, and receiver operating characteristic (ROC) curves are needed to evaluate its utility as a screening tool.
2. It identifies modifiable lifestyle factors—BMI, smoking, and physical activity—as critical targets for prevention.
3. All the statistical models were adjusted for major potential confounders.
4. Physical activity was assessed using a validated standardized questionnaire (IPAQ) administered by trained professionals.

### 5.2. Limitations

1. Self-reported dietary data from FFQs may be subject to recall or social desirability bias, although participants were encouraged to report accurately.
2. Alcohol intake was excluded from the LIS because of negligible consumption, which might affect construct validity. Although consumption was negligible in this population, including this variable could introduce noise into the score.
3. Despite adjustments for several covariates, residual confounding from unmeasured variables cannot be ruled out.
4. The LIS was originally validated in a U.S. population, and its direct application to the Mexican population may not fully capture cultural or environmental differences. Nonetheless, studies in diverse populations have reported consistent associations. Additionally, the results pertain to women attending the IMSS in Mexico City, so their applicability to other populations should be considered with caution.
5. The LIS was constructed on the basis of physical activity and smoking status reported at the end of pregnancy, which focuses on habits during pregnancy, so this does not allow us to make causal inferences, since it might have occurred simultaneously with the diagnosis of GDM.
6. We did not evaluate diet qualitatively, such as dietary patterns or the Dietary Inflammatory Index, which might be more appropriate.
7. Adiponectin was included to assess its effects on LIS and GDM and to identify which metabolic and inflammatory pathways may mediate these effects. This could underestimate the model’s total effect, so the model should be considered an adjusted association, rather than a causal effect. When we removed adiponectin from the regression model, the OR for the highest LIS decreased from (OR 3.723, CI 95%: 1.191–11.640, *p* = 0.024) to (OR 3.033, CI 95%: 1.108–8.302, *p* = 0.031). Therefore, adjustment for adiponectin does not underestimate the effect of LIS.
8. Another limitation is that we did not use a specific inflammatory biomarker, such as TNF-α, to validate the LIS. However, it was used to construct the β coefficients of the original LIS.
9. Although we do not have inter-rater reliability, all participating health professionals were standardized and used valid measurements and questionnaires.
10. We do not have the intra- or interassay coefficient of variation, since calculation was only performed once.

### 5.3. Implications and Future Directions

The present findings support the use of the Lifestyle Inflammation Score as a screening and monitoring tool for women during pregnancy and in the postpartum period. Integrating LIS into clinical practice could help stratify risk and prioritize preventive strategies, particularly weight management, smoking cessation, and physical activity promotion, to mitigate the progression from GDM to GMA and, ultimately, to T2D.

Future research should validate the LIS in larger, ethnically diverse populations and explore its integration with other inflammatory and metabolic biomarkers to improve its predictive accuracy.

## 6. Conclusions

A higher Lifestyle Inflammation Score (LIS), reflecting more adverse lifestyle exposures, significantly increased the risk of developing gestational diabetes mellitus (GDM) and glucose metabolism alterations (GMA) postpartum. These findings highlight the role of low-grade inflammation in the metabolic trajectory from pregnancy to later life, underscoring the importance of preventive strategies targeting modifiable factors—namely, BMI, smoking, and physical inactivity.

Early identification of women with a proinflammatory lifestyle through the LIS may enable timely, personalized interventions to improve maternal health outcomes and reduce the risk of long-term metabolic complications.

## Figures and Tables

**Table 1:** Components of the LIS, rationales for inclusion, and *β* coefficients.

LIS Components ^1^	Rationale for Inclusion	*β* Coefficients
Alcohol consumption	Heavy drinker: >14 g of ethanol/day for women vs. nondrinker	0.30
	Moderate drinker: 14 g of ethanol/day for women vs. non-drinker	−0.66
Physical activity	Moderately physically active: exercises 1–3 times a week vs. does not exercise	−0.18
	Heavily physically active: exercises ≥4 times a week vs. does not exercise	−0.41
Current smoker	Currently smokes tobacco vs. does not currently smoke tobacco	0.50
Overweight and obese BMI	Overweight BMI vs. normal BMI	0.89
	Obese BMI vs. normal BMI	1.57

^1^ All lifestyle components were dummy variables, coded as “1” for the non-referent category and “0” for the referent category.

**Table 2:** Clinical and Anthropometric Parameters by Tertiles of the Lifestyle Inflammation Score in the Total Population.

	LIS T1 *n* = 120 Median (IQR)	LIS T2 *n* = 127 Median (IQR)	LIS T3 *n* = 131 Median (IQR)	*p*	Effect Size
At the end of pregnancy (38 gestational weeks)					
Age (years)	32 (26.2–35)	33 (29–36)	33 (29–37)	0.053	0.010 ^†^
Family history of diabetes, *n* (%) *	87 (72.5)	91 (71.7)	107 (81.7)	0.086	0.107 ^††^
Cesarean section delivery, *n* (%) *	89 (74.2)	99 (78)	97 (74)	0.966	0.042 ^††^
Gravidity	2 (1–3)	2 (1–3)	2 (1–3)	0.128	0.005 ^†^
Apgar	8 (8–8.7)	8 (8–9)	8 (8–8)	0.644	<0.001 ^†^
Pregestational BMI (kg/m^2^)	23.3 (21.6–24.2)	27.4 (26.1–28.8)	32.8 (31–35)	**<0.001**	**0.763** ^†^
At the end of pregnancy BMI (kg/m^2^)	26.6 (25.1–29.5)	30.8 (29.1–32.1)	34.6 (31.7–37.8)	**<0.001**	**0.464** ^†^
Physical Activity, *n* (%) *				**0.001**	0.208 ^††^
Physically inactive	32 (26.7)	36 (28.3)	40 (30.5)		
Moderately active	63 (52.5)	83 (65.4)	90 (68.7)		
Heavily active	25 (20.8)	8 (6.3)	1 (0.8)		
Actively smoking, *n* (%) *	8 (6.7)	1 (0.8)	32 (24.4)	**<0.001**	**0.327** ^††^
Postpartum					
Weight gain during pregnancy (Kg)	10 (6–14.3)	8 (4–12)	4 (0.7, 8)	**<0.001**	0.149 ^†^
Postpartum BMI **	24.2 ± 3.7	27.9 ± 3.0	33.3 ± 4.3	**<0.001**	**0.541** ^†^
Fat mass (%)	30.8 (28.3–33.5)	36.0 (33.2–39.1)	42.5 (38.7–45.4)	**<0.001**	**0.512** ^†^
Waist/Hip ratio **	0.83 ± 0.05	0.86 ± 0.06	0.87 ± 0.06	**<0.001**	0.086 ^†^
Systolic blood pressure (mmHg)	108.5 (100–113)	110 (104–118)	112 (110–120)	**<0.001**	0.072 ^†^
Diastolic blood pressure (mmHg)	70 (65–76.7)	72 (68–80)	74 (70–80)	**<0.001**	0.038 ^†^
Exclusive breastfeeding, *n* (%) *	79 (65.8)	81 (63.8)	74 (56.5)	0.125	0.083 ^††^

Kruskal–Wallis test, * Linear-by-linear association test, ** Mean (SD), one-way ANOVA test, *p* < 0.05; ^†^ *η*^2^ for continuous variables; ^††^ Cramér’s V for categorical variables. Negative *η*^2^ were truncated to <0.001; LIS: Lifestyle Inflammatory Score, T: Tertile, IQR: Interquartile Range, BMI: Body Mass Index, physical activity per IPAQ manual. Values in bold are statistically significant (*p* < 0.05).

**Table 3:** Biochemical Parameters by Tertiles of the Lifestyle Inflammation Score in the Total Population.

	LIS T1 *n* = 120 Median (IQR)	LIS T2 *n* = 127 Median (IQR)	LIS T3 *n* = 131 Median (IQR)	*p*	Effect Size
At the end of pregnancy (38 gestational weeks)					
Fasting serum glucose (mg/dL)	67.5 (56–84.7)	67 (59–78)	69 (57–88)	0.706	<0.001 ^†^
Triglycerides (mg/dL)	318.5 (256.2–401)	321 (275–407)	340 (284–416)	0.156	0.005 ^†^
LDL cholesterol (mg/dL)	144.8 (110.6–171.9)	135 (111.4–167.7)	130.4 (105.1–163.4)	0.310	0.001 ^†^
HDL cholesterol (mg/dL)	54.5 (47–65)	54 (46–62)	53 (42–62)	0.313	0.001 ^†^
Uric acid (mg/dL)	5 (4.3–5.9)	5.1 (4.5–5.8)	5.2 (4.7–6.1)	0.127	0.006 ^†^
Fasting serum Insulin (U/mL)	10.2 (6.8–16.8)	10.2 (6.7–17.1)	13.2 (8.7–26.5)	**0.002**	0.027 ^†^
HbA1c (%)	5.4 (5.2–5.8)	5.6 (5.3–5.8)	5.6 (5.2–6)	**0.015**	0.017 ^†^
HOMA-IR	1.5 (0.9–3.4)	1.7 (0.9–3.1)	2.1 (1.2–5.6)	**0.015**	0.017 ^†^
Leptin (pg/mL)	7088.4 (4918.3–8741.8)	6921.6 (5399.3–8542.2)	7279.6 (4920.8–8710.2)	0.985	<0.001 ^†^
Adiponectin (pg/mL)	2956.8 (2404.2–3744.1)	3306 (2448.5–3828.8)	3402.5 (2736.1–4100.7)	**0.005**	0.023 ^†^
Carbonylated proteins (nmol/mL)	25.6 (17.5–30.9)	28.6 (19–35.4)	29.5 (25.2–34.7)	**0.002**	0.028 ^†^
Postpartum					
Fasting serum glucose (mg/dL)	84 (76.2–91.7)	86 (77–94)	92 (82–101)	**<0.001**	0.057 ^†^
Triglycerides (mg/dL)	91 (69.2–152)	117 (86–187)	143 (109–198)	**<0.001**	0.079 ^†^
LDL cholesterol (mg/dL)	104.7 (88.9–122.6)	114.2 (97.5–135)	114.6 (101–132.5)	**0.014**	0.017 ^†^
HDL cholesterol (mg/dL)	52 (44–60.5)	49 (42–56)	46 (39–53)	**<0.001**	0.040 ^†^
Uric acid (mg/dL)	4.5 (4–5.3)	5 (4.4–5.7)	5.4 (4.7–6)	**<0.001**	0.076 ^†^
Fasting serum insulin (U/mL)	6.1 (4.4–8.7)	8.7 (5.4–12.4)	10.9 (7.8–16.9)	**<0.001**	0.138 ^†^
HbA1c (%)	5.3 (5.1–5.6)	5.5 (5.2–5.8)	5.6 (5.3–6)	**<0.001**	0.041 ^†^
HOMA-IR	1.2 (0.8–1.8)	1.6 (0.9–2.7)	2.6 (1.7–4.3)	**<0.001**	0.141 ^†^

Kruskal–Wallis test, *p* < 0.05, ^†^ *η*^2^ for continuous variables. Negative *η*^2^ were truncated to <0.001; LIS: Lifestyle Inflammatory Score, T: Tertile, IQR: Interquartile Range, LDL cholesterol: Low-density-level cholesterol, HDL cholesterol: High-density-level cholesterol, HbA1c: Glycated Hemoglobin A1c, HOMA-IR: The Homeostasis Model Assessment of Insulin Resistance. Values in bold are statistically significant (*p* < 0.05).

**Table 4:** Postpartum Dietary Parameters by Tertiles of the Lifestyle Inflammation Score in the Total Population.

	LIS T1 *n* = 120 Median (IQR)	LIS T2 *n* = 127 Median (IQR)	LIS T3 *n* = 131 Median (IQR)	*p*	Effect Size
Energy (Kcals)	2157.7 (1682.6–2655.1)	2083.2 (1619.1–2635.9)	1829.2 (1495.7–2329.3)	**0.009**	<0.020 ^†^
Proteins (% TE)	14.0 (12.7–15.3)	14.2 (12.5–15.7)	14.5 (13.3–15.9)	0.127	0.006 ^†^
Carbohydrates (% TE) *	50.2 ± 7.4	49.2 ± 7.8	49.2 ± 7.2	0.531	<0.001 ^†^
Fats (% TE)	36.2 (32.6–40.2)	37 (32–42.4)	36.6 (32.7–40.8)	0.684	<0.001 ^†^
Saturated fatty acids (% TE)	10.8 (9.5–12.2)	11 (9.7–12)	11.1 (9.6–12.5)	0.798	<0.001 ^†^
Monounsaturated fatty acids (% TE)	14.7 (12.9–16.8)	14.2 (12.6–16.7)	14.8 (12.5–16.9)	0.929	<0.001 ^†^
Polyunsaturated fatty acids (% TE)	6.4 (5.6–8.1)	6.8 (5.6–9.6)	6.7 (6–8.4)	0.326	0.001 ^†^

Kruskal–Wallis test, * Mean (SD), one-way ANOVA test, *p* < 0.05, ^†^ *η*^2^ for continuous variables. Negative *η*^2^ was truncated to <0.001; LIS: Lifestyle Inflammatory Score, T: Tertile, IQR: Interquartile Range, TE: Total Energy. Values in bold are statistically significant (*p* < 0.05).

**Table 5:** Multivariate Logistic Regression Analysis of Gestational Diabetes Mellitus.

Variable	OR	95% CI	*p*
LIS (T1)	Reference	-	-
LIS (T2)	2.004	0.667–6.023	0.216
LIS (T3)	3.723	1.191–11.640	0.024

Model adjusted for: family history of diabetes, gestational weight gain, carbonylated proteins, and adiponectin. Model fit: *r* = 0.849; *p* < 0.001.

**Table 6:** Clinical and Anthropometric Parameters During Pregnancy and Postpartum by Lifestyle Inflammation Score Tertiles.

	LIS T1 *n* = 61 Median (IQR)	LIS T2 *n* = 90 Median (IQR)	LIS T3 *n* = 95 Median (IQR)	*p*
At the end of pregnancy (38 gestational weeks)				
Age (years)	33 (28.5–38)	34 (30–37)	34 (31–37)	0.528
Family history of diabetes, *n* (%) *	48 (78.7)	69 (76.7)	77 (81.1)	0.664
Cesarean section delivery, *n* (%) *	44 (72.1)	69 (76.7)	71 (74.7)	0.769
Gravidity	2 (1–3)	2 (1–3)	2 (1–3)	0.414
Apgar	8 (8–8.5)	8 (8–9)	8 (8–8)	0.500
Pregestational BMI (kg/m^2^)	23.6 (22.1–24.4)	27.7 (26.5–29.2)	33.4 (31.2–35.9)	**<0.001**
At the end of pregnancy BMI (kg/m^2^)	26.4 (25.1–29.0)	30.8 (29.3–32.5)	35.2 (32.4–38.2)	**<0.001**
Physical Activity, *n* (%) *				
Physically inactive	17 (27.9)	25 (27.8)	32 (33.7)	**0.001**
Moderately active	27 (44.3)	58 (64.4)	62 (65.3)	
Heavily active	17 (27.9)	7 (7.8)	1 (1.1)	
Actively smoking, *n* (%) *	0 (0)	7 (7.8)	20 (21.1)	**<0.001**
Pharmacological treatment during pregnancy, *n* (%) *	23 (41.1)	41 (47.7)	55 (59.1)	**0.027**
Postpartum				
Weight gain during pregnancy (kg)	9 (4.7–13)	8 (4.3–12.5)	3 (−1, 8)	**<0.001**
Postpartum BMI **	24.9 ± 4.1	28.5 ± 3.0	33.6 ± 4.4	**<0.001**
Fat mass (%)	32.3 (29.0–35.3)	36.8 (34.5–39.6)	43.1 (39.4–45.6)	**<0.001**
Waist/Hip ratio **	0.83 ± 0.05	0.86 ± 0.05	0.88 ± 0.05	**<0.001**
Systolic blood pressure (mmHg)	110 (103–114.5)	111.2 (104.7–119.2)	114 (110–120)	**0.001**
Diastolic blood pressure (mmHg)	70 (65–78)	74.7 (70–80)	74 (70–80)	**0.004**
Exclusive breastfeeding, *n* (%) *	38 (62.3)	54 (60)	54 (56.8)	0.490

Kruskal–Wallis test, * Linear-by-linear association test, ** Mean (SD), one-way ANOVA test, *p* < 0.05; LIS: Lifestyle Inflammatory Score, T: Tertile, IQR: Interquartile Range, BMI: Body Mass Index, physical activity per IPAQ manual. Values in bold are statistically significant (*p* < 0.05).

**Table 7:** Biochemical Parameters During Pregnancy and Postpartum by Tertiles of the Lifestyle Inflammation Score.

	LIS T1 *n* = 61 Median (IQR)	LIS T2 *n* = 90 Median (IQR)	LIS T3 *n* = 95 Median (IQR)	*p*
At the end of pregnancy (38 gestational weeks)				
Fasting serum glucose (mg/dL)	73 (62.5–92)	70 (62–82)	70 (60–91)	0.504
Triglycerides (mg/dL)	333 (272–418)	338 (281.2–444.7)	345 (277–410)	0.719
LDL cholesterol (mg/dL)	131.7 (104.8–167.4)	142.1 (112.1–167.6)	131.3 (102.6–163.4)	0.723
HDL cholesterol (mg/dL)	53 (45–61)	55 (44–64.2)	52 (41–61)	0.185
Uric acid (mg/dL)	5.1 (4.1–6.1)	5 (4.4–5.8)	5.3 (4.8–6.1)	**0.048**
Fasting serum Insulin (U/mL)	11.3 (6.8–21.4)	11.5 (7.3–21.6)	13.6 (9.1–28.7)	0.112
HbA1c (%)	5.5 (5.3–5.8)	5.7 (5.4–6)	5.7 (5.4–6.0)	0.096
HOMA-IR	1.8 (1.1–5.0)	1.9 (1.0–4.2)	2.1 (1.4–7.3)	0.263
Leptin (pg/mL)	5620.1 (3548.1–7909.9)	6303.1 (4590.5–8048)	6831.2 (4500.6–8179.6)	0.174
Adiponectin (pg/mL)	3516.7 (2956.8–4058.9)	3574 (2784.7–4146.2)	3696.9 (3211.1–4187.6)	0.323
Carbonylated proteins (nmol/mL)	30.9 (26.5–36.4)	32.9 (27.8–38.4)	31.5 (26.8–36.3)	0.256
Postpartum				
Fasting serum glucose (mg/dL)	90 (81.5–95.5)	90 (80.7–99)	95 (87–102)	**0.007**
Glucose 2-h (mg/dL) post OGTT	102 (85.2–124.5)	116 (92.7–143.1)	115.5 (99–143.1)	**0.016**
Triglycerides (mg/dL)	121 (75.5–181.5)	142.5 (94.7–208.2)	141 (105–195)	**0.033**
LDL cholesterol (mg/dL)	107.6 (86–121.5)	114.7 (100.2–135)	118.7 (102.7–132.5)	**0.028**
HDL cholesterol (mg/dL)	49 (39.5–60)	47 (40–54)	46 (38–52)	0.111
Uric acid (mg/dL)	4.6 (4–5.3)	5.1 (4.5–5.7)	5.4 (4.7–6)	**<0.001**
Fasting serum insulin (U/mL)	6.4 (4.6–10.3)	9.5 (5.8–13)	11.2 (7.8–17)	**<0.001**
Insulin 2-h (U/mL) post OGTT	34.5 (16.1–50.8)	44.1 (28.9–66.1)	58.1 (28.3–80)	**0.001**
HbA1c (%)	5.6 (5.3–5.8)	5.6 (5.4–5.8)	5.8 (5.4–6.1)	0.094
HOMA-IR	1.4 (1–2.2)	2 (1.3–3)	2.8 (1.7–4.6)	**<0.001**

Kruskal–Wallis test, *p* < 0.05; LIS: Lifestyle Inflammatory Score, T: Tertile, IQR: Interquartile Range, LDL cholesterol: Low-density-level cholesterol, HDL cholesterol: High-density-level cholesterol, OGTT: Oral Glucose Tolerance Test; HbA1c: Glycated Hemoglobin A1c, HOMA-IR: The Homeostasis Model Assessment of Insulin Resistance. Values in bold are statistically significant (*p* < 0.05).

**Table 8:** Postpartum Dietary Parameters by Tertiles of the Lifestyle Inflammation Score.

	LIS T1 *n* = 61 Median (IQR)	LIS T2 *n* = 90 Median (IQR)	LIS T3 *n* = 95 Median (IQR)	*p*
Energy (kcals)	2041.9 (1599.6–2526.6)	1994.4 (1594.1–2538.9)	1896.3 (1515.2–2310.1)	0.363
Proteins (% TE)	14.6 (13.3–16)	14.6 (12.7–15.9)	14.5 (13.3–16.1)	0.698
Carbohydrates (% TE) *	48.9 ± 7.3	48.3 ± 8.2	49.5 ± 7.4	0.550
Fats (% TE)	36.6 (34.7–40.7)	37.7 (32.2–43.4)	35.9 (32.2–40.1)	0.255
Saturated fatty acids (% TE)	11.6 (10.1–12.6)	11.2 (9.9–12.5)	11 (9.6–12.3)	0.550
Monounsaturated fatty acids (% TE)	15.1 (13.6–17)	14.4 (12.9–16.7)	14.6 (12.4–16.9)	0.301
Polyunsaturated fatty acids (% TE)	6.5 (5.6–7.5)	6.8 (5.7–9.9)	6.8 (6–8.7)	0.229

Kruskal–Wallis test, * Mean (SD), one-way ANOVA test, *p* < 0.05; LIS: Lifestyle Inflammatory Score, T: Tertile, IQR: Interquartile Range, TE: Total Energy.

**Table 9:** Multivariate Logistic Regression Analysis of Glucose Metabolism Alterations.

Variable	OR	95% CI	*p*
LIS (T1)	Reference	-	-
LIS (T2)	1.836	0.880–3.830	0.105
LIS (T3)	2.685	1.253–5.755	0.011

Model adjusted for: pharmacological treatment during pregnancy, gestational weight gain, and exclusive breastfeeding. Model fit: *r* = 0.102; *p* = 0.044.

## Data Availability

The data supporting this study are available upon reasonable request from the corresponding author. Data sharing is restricted as the study is still ongoing, and premature release of the dataset could affect its completion.

## Abbreviations

IMSS	Instituto Mexicano del Seguro Social
GMA	Glucose Metabolism Alterations
GDM	Gestational Diabetes Mellitus
LIS	Lifestyle Inflammation Score
BMI	Body Mass Index
T2D	Type 2 Diabetes
LGI	Low-Grade Inflammation
CRP	C-Reactive Protein
IPAQ	International Physical Activity Questionnaire
T	Tertile
OGTT	Oral Glucose Tolerance Test
FSG	Fasting Serum Glucose
FFQ	Food Frequency Questionnaire
IQR	Interquartile Range
TE	Total Energy
HOMA-IR	Homeostasis Model Assessment of Insulin Resistance
